# Environmental Mycotoxins and Brain Health: Protective Role of Bromelain Against Fumonisin B1 in SH‐SY5Y Cells

**DOI:** 10.1002/tox.70059

**Published:** 2026-02-24

**Authors:** Gianluca Antonio Franco, Francesca Inferrera, Nicla Tranchida, Davide Di Paola, Roberta Fusco, Salvatore Cuzzocrea, Enrico Gugliandolo

**Affiliations:** ^1^ Department of Veterinary Science University of Messina Messina Italy; ^2^ Department of Chemical, Biological, Pharmaceutical and Environmental Science University of Messina Messina Italy; ^3^ Link University, Rome Campus Rome Italy

**Keywords:** apoptosis, bromelain, fumonisin B1, inflammation, mitochondrial dysfunction, neurotoxicity, oxidative stress, SH‐SY5Y

## Abstract

Fumonisin B1 (FB1), a mycotoxin commonly found in contaminated food and feed, has been increasingly implicated in neurotoxicity, although its mechanisms remain poorly understood. This study investigates the neurotoxic potential of FB1 in human SH‐SY5Y neuroblastoma cells, both undifferentiated and RA‐differentiated, and evaluates the protective effect of bromelain, a natural proteolytic enzyme with antioxidant and anti‐inflammatory properties. Cells were exposed to 50 μM FB1 for 24 h, with or without co‐treatment with bromelain (10 or 50 μg/mL). FB1 significantly reduced cell viability and triggered reactive oxygen species (ROS) production, mitochondrial membrane depolarization, lipid peroxidation, inflammatory cytokine release (IL‐6 and TNF‐α), and apoptosis, particularly in differentiated cells. Bromelain co‐treatment attenuated these effects in a dose‐dependent manner, preserving mitochondrial function, reducing oxidative and inflammatory markers, and lowering apoptotic cell death. These findings emphasize the neurotoxic risk posed by FB1 and highlight bromelain as a promising multi‐target protective agent. Identifying effective countermeasures against environmental neurotoxins is crucial for public health and disease prevention.

## 1 Introduction

### 1.1 Background on Fumonisin B1 and Neurotoxicity

Fumonisins are a group of mycotoxins primarily produced by Fusarium verticillioides and Fusarium proliferatum, commonly contaminating maize‐based food and feed worldwide. Among them, fumonisin B1 (FB1) is the most prevalent and toxic. FB1 exerts its toxic effects by disrupting sphingolipid metabolism, leading to various pathological outcomes, including hepatotoxicity, nephrotoxicity, and carcinogenicity. Although most research has historically focused on liver and kidney toxicity, emerging evidence suggests that FB1 may also affect the nervous system, raising concerns about its neurotoxic potential, especially in vulnerable populations [1]. Despite these concerns, relatively little is known about the direct neurotoxic mechanisms of FB1, particularly in human‐derived neuronal models. FB1 has been classified as an emerging mycotoxin of concern for both human and animal health, due to its widespread occurrence, potential cumulative exposure, and limited regulation in some regions. Its capacity to cross biological barriers and to interfere with cellular signaling pathways further supports the need for in‐depth investigation of its neurotoxic profile [2]. The central nervous system (CNS) is particularly vulnerable to xenobiotics capable of generating oxidative stress, due to its high oxygen demand, elevated lipid content, and relatively low antioxidant defenses [3].

### 1.2 Comparative Mechanisms With Other Mycotoxins

Beyond fumonisin B1, several agriculturally relevant mycotoxins threaten neural health through partially overlapping yet mechanistically distinct pathways [4]. Ochratoxin A, produced by Aspergillus and Penicillium species, rapidly triggers DNA damage, reactive‐oxygen‐species accumulation and caspase‐driven apoptosis in neuronal cells at low‐micromolar doses [5]. Zearalenone, an oestrogenic toxin from Fusarium species, provokes lipid peroxidation and redox imbalance, although its impact on cell viability is comparatively modest [6]. Deoxynivalenol can traverse the blood–brain barrier and activate MAPK and pyroptotic signaling; nanomolar to micromolar doses disrupt mitochondrial bioenergetics in primary neurons [7]. The type‐A trichothecene T‐2 toxin is exceptionally potent, inducing mitochondrial depolarisation and oxidative DNA damage at sub‐micromolar levels [8]. These mechanistic differences highlight the need for dedicated studies on FB1‐mediated neuronal stress and for tailored mitigation strategies.

### 1.3 Role of Oxidative Stress and Inflammation in Neuronal Injury

From a One Health perspective, these toxins co‐occur in maize‐based food and feed, posing cumulative risks to humans, livestock and companion animals while also impacting environmental health through contaminated agricultural by‐products; understanding FB1's unique neuro‐toxic profile is therefore essential for integrated risk assessment and the development of cross‐species mitigation strategies. Reactive oxygen species (ROS)‐inducing agents like FB1 [9] can profoundly affect neuronal homeostasis, leading to oxidative damage to membranes, proteins, and DNA. Oxidative stress is recognized as a key contributor to biochemical disturbances in the brain. It arises when the generation of reactive oxygen species (ROS) surpasses the capacity of endogenous antioxidant defense systems. Due to its high oxygen demand and abundance of polyunsaturated lipids, the brain is particularly prone to oxidative damage. Such redox imbalance can significantly impair central nervous system (CNS) functions and contribute to neurological dysfunction. While oxidative stress has traditionally been associated with the pathogenesis of neurodegenerative diseases such as Alzheimer's, Parkinson's, and Huntington's disease, recent evidence also implicates it in neuropsychiatric conditions, including anxiety and depressive disorders [10]. In the context of FB1 exposure, this redox imbalance is believed to play a pivotal role in triggering neuroinflammatory cascades and mitochondrial dysfunction, both of which are key contributors to neuronal injury [11]. Prolonged or excessive oxidative stress in the CNS may result in chronic neuroinflammation, glial activation, and ultimately neurodegeneration, all of which are hallmarks of several neurological disorders, including Alzheimer's and Parkinson's disease [12, 13].

### 1.4 Rationale for Bromelain

Given the increasing awareness of the role of environmental toxins in the onset or progression of neurodegenerative conditions, it is critical to understand how emerging mycotoxins like FB1 interact with neuronal cells and affect their redox and inflammatory balance. In this context, the One Health perspective—which emphasizes the interdependence of human, animal, and environmental health—is particularly relevant. Mycotoxins such as FB1 affect not only human populations through contaminated food products, but also animals, particularly livestock and companion species, via contaminated feed. The identification of effective protective strategies against FB1‐induced neurotoxicity therefore has cross‐species relevance, contributing to both public health and veterinary safety. Given the central role of oxidative stress in the pathogenesis of various neurological disorders, significant efforts have been directed toward the development of antioxidant‐based therapeutic strategies. Although only a few antioxidant agents—such as edaravone, idebenone, and dimethyl fumarate—have been approved for clinical use in neurological conditions, many others are currently being evaluated in preclinical and clinical studies.

Antioxidants can function through different mechanisms, acting either as direct scavengers of reactive species or by indirectly enhancing endogenous antioxidant defenses. It is important to note that the aim of antioxidant therapy is not to eliminate ROS altogether, but rather to restore redox homeostasis in pathological states where ROS levels are abnormally elevated. The brain's heightened vulnerability to oxidative stress, due to its metabolic profile, makes redox imbalance a particularly relevant therapeutic target. However, the limited efficacy of some direct antioxidants may stem from their inability to neutralize highly compartmentalized ROS or from insufficient bioavailability. For this reason, increasing attention has been paid to compounds with pleiotropic antioxidant activity, capable of modulating broader cellular defense pathways—such as those regulating gene expression and anti‐inflammatory signaling [13]. In this regard, bromelain represents a promising candidate, as it not only exhibits direct and indirect antioxidant effects but also modulates inflammatory mediators and apoptotic pathways. Its ability to act on multiple targets, combined with its established safety profile and natural origin, makes it an attractive agent for further investigation in the context of neuroprotection against mycotoxin‐induced oxidative injury [14]. Bromelain, a mixture of proteolytic enzymes extracted from the pineapple stem ( *Ananas comosus* ), has been widely used in both human and veterinary medicine for its anti‐oedematous, anti‐inflammatory, and analgesic properties. Bromelain has shown potential in modulating oxidative stress, reducing the expression of pro‐inflammatory cytokines, and limiting apoptotic signaling, yet its neuroprotective effects against mycotoxin‐induced damage remain unexplored [15]. Its well‐documented antioxidant and anti‐inflammatory properties make bromelain a promising candidate for neuroprotection, especially in contexts involving redox imbalance and inflammation‐induced damage [16]. Furthermore, recent studies have begun to explore its potential neuroprotective effects, highlighting both its multifunctional bioactivity and its favorable safety profile, which support its applicability in preventive or therapeutic strategies targeting neurotoxicity and neurodegeneration [16]. Interestingly, bromelain has also been shown to reduce both inflammation and oxidative stress through direct mechanisms, for example by enhancing the activation of the Nrf2 signaling pathway [17].

### 1.5 Aim of the Study

Therefore, the aim of this study was to investigate the neurotoxic effects of FB1 in both undifferentiated and RA‐differentiated SH‐SY5Y human neuroblastoma cells, and to evaluate the protective potential of bromelain. This work was conceived as a proof‐of‐concept study aimed at elucidating the mechanistic basis of FB1‐induced neurotoxicity and the potential protective action of bromelain in a controlled in vitro model, focused on key early events in neuronal injury, including ROS generation, mitochondrial membrane depolarization, lipid peroxidation, cytokine release, and apoptosis, to provide novel insight into the mechanisms of FB1 neurotoxicity and the efficacy of bromelain as a multi‐target protective agent in vitro.

## 2 Materials and Methods

### 2.1 Cell Culture and Differentiation

Human neuroblastoma SH‐SY5Y cells (ATCC, CRL‐2266) were cultured in Dulbecco's Modified Eagle Medium/Nutrient Mixture F‐12 (DMEM/F12, Gibco) supplemented with 10% fetal bovine serum (FBS), 1% penicillin–streptomycin, and maintained at 37°C in a humidified incubator with 5% CO_2_. For neuronal differentiation, SH‐SY5Y cells were seeded at 5 × 10^4^ in 12 multi well plate, after 24 h, exposed to 10 μM all‐trans retinoic acid (RA) (Sigma‐Aldrich, R2625). Differentiation was carried out for 7 consecutive days in DMEM/F‐12 containing 1% FBS, with complete medium (including RA) replaced every 48 h according to validated protocols [18, 19]. Cells were used for treatments immediately after the 7‐day RA differentiation period without further passaging.

### 2.2 Treatments

Cells were exposed to fumonisin B1 (FB1) at 50 μM dissolved in complete medium for 24 h to induce neurotoxicity [20]. To assess potential neuroprotection, cells were co‐treated with bromelain at final concentrations of 10 or 50 μg/mL in accordance with previously published studies [16]. Control cells received vehicle only. Bromelain was purchased from Sigma‐Aldrich (Cat# B5144). For treatments, working solutions were freshly diluted in complete culture medium; a bromelain‐alone control of 10 or 50 μg/mL and a vehicle control were included in every experiment.

### 2.3 Cell Viability Assay (MTT)

Cell viability was assessed using the MTT (3‐(4,5‐dimethylthiazol‐2‐yl)‐2,5‐diphenyltetrazolium bromide) reduction assay, as previously described with minor modifications Gugliandolo et al. [21]. SH‐SY5Y cells (undifferentiated and RA‐differentiated) were seeded in 96‐well plates at a density of 4 × 10^4^ cells/well in 150 μL of complete DMEM/F12 and incubated for 24 h at 37°C in a 5% CO_2_ atmosphere. After treatments with FB1 (50 μM), with or without bromelain (10 or 50 μg/mL) for 24 h, the culture medium was removed and replaced with 150 μL of MTT solution (0.5 mg/mL in DMEM/F12), freshly prepared and protected from light. Cells were incubated with MTT for 3 h at 37°C to allow formazan crystal formation. After incubation, the MTT solution was discarded, and 150 μL of DMSO was added to each well to solubilize the formazan crystals. The plate was gently shaken for 10 min to ensure complete dissolution. Absorbance was measured at 570 nm using a Multiskan FC microplate reader (Thermo Fisher Scientific). Cell viability was expressed as a percentage relative to the untreated control group.

### 2.4 Reactive Oxygen Species (ROS) Detection

Intracellular ROS production was evaluated using the fluorescent probe carboxy‐H₂DCFDA (Invitrogen), which detects general oxidative stress. Following treatments, SH‐SY5Y cells were incubated with 10 μM carboxy‐H₂DCFDA diluted in serum‐free medium for 30 min at 37°C in the dark. Cells were then washed with PBS and immediately analyzed by flow cytometry using the Attune NxT Acoustic Focusing Cytometer (Thermo Fisher Scientific). Mean fluorescence intensity (MFI) was recorded and used as a quantitative index of ROS levels. For qualitative assessment, fluorescence images were captured using an EVOS M5000 Imaging System (Thermo Fisher Scientific) under GFP‐compatible filter settings.

### 2.5 Mitochondrial Membrane Potential (Δψm)

Mitochondrial membrane potential was assessed using Rhodamine 123 (Sigma‐Aldrich), a cationic dye that accumulates in active mitochondria. After treatment, cells were incubated with 10 μM Rhodamine 123 for 20 min at 37°C, protected from light. Cells were then washed with PBS and immediately analyzed by flow cytometry on the Attune NxT Cytometer. A decrease in fluorescence intensity was interpreted as a loss of Δψm, indicating mitochondrial depolarization.

### 2.6 Immunofluorescence Nrf2

SH‐SY5Y cells, either undifferentiated or differentiated, were seeded in sterile 48‐well plates. Following treatments for 24 h, cells were fixed in 4% paraformaldehyde in PBS for 10 min at room temperature and rinsed three times with PBS. Permeabilization was performed with 0.1% Triton X‐100 in PBS for 5 min, followed by blocking in 5% bovine serum albumin (BSA) in PBS for 30 min. Cells were incubated overnight at 4°C with a rabbit monoclonal antibody against Nrf2 (sigma, SAB4501984) and Nfkb (proteintech10745‐1‐AP) diluted in PBS containing 1% BSA. After washing, cells were incubated with an Alexa Fluor 488‐conjugated goat anti‐rabbit IgG secondary antibody (Invitrogen, Thermo Fischer) or Goat anti‐Rabbit IgG Secondary Antibody, Texas Red for 1 h at room temperature in the dark. Nuclei were counterstained with 4′,6‐diamidino‐2‐phenylindole (DAPI, 1 μg/mL, 5 min) and imaged using an EVOS M5000 fluorescence microscope (Thermo Fisher Scientific). For quantification, images were analyzed using ImageJ/FIJI software. Nuclear regions of interest (ROI) were defined by DAPI staining, while cytoplasmic ROIs were obtained by subtracting nuclear masks from whole‐cell areas. The mean fluorescence intensity of Nrf2 was measured in both compartments, and the nuclear‐to‐cytoplasmic (N/C) fluorescence ratio was calculated for each cell. At least 50 cells per condition were analyzed across three independent experiments.

### 2.7 Lipid Peroxidation (MDA Assay)

Lipid peroxidation was assessed by quantifying malondialdehyde (MDA) levels using the thiobarbituric acid reactive substances (TBARS) assay, which detects MDA as an index of oxidative damage to membrane lipids. After treatment, SH‐SY5Y cells were washed with PBS and lysed in RIPA buffer containing protease inhibitors. Lysates were centrifuged at 12 000*g* for 10 min at 4°C, and the supernatants were collected for analysis. Cell lysate and TBA reagent were mixed and incubated at 95°C for 60 min in a dry block heater. After cooling on ice for 10 min, the samples were centrifuged at 10 000*g* for 10 min to remove debris. The absorbance of the resulting pink‐colored MDA‐TBA adduct was measured at 532 nm using a UV–Vis spectrophotometer with quartz cuvettes. MDA concentrations were calculated from a standard curve generated with 1,1,3,3‐tetramethoxypropane (TMP) and normalized to total protein content (Bradford assay). Results were expressed as MDA/mg protein.

### 2.8 RT‐PCR

Total RNA was extracted from SH‐SY5Y cells using the RNeasy Mini Kit (Qiagen) according to the manufacturer's instructions. RNA concentration and purity were determined spectrophotometrically nanodrop; for cDNA synthesis, RNA was reverse‐transcribed using the iScript cDNA Synthesis Kit (Bio‐Rad, Hercules, CA, USA). Quantitative real‐time PCR was performed on a CFX96 Real‐Time PCR Detection System (Bio‐Rad) using the QuantiTec SYBR Green PCR Kit (Qiagen) with QuantiTec Primer Assays (Qiagen) specific for the target genes (HO‐1). A melt‐curve analysis was performed at the end of the amplification protocol to verify the specificity of PCR products. Relative gene expression was calculated using the 2^−^ΔΔCt method, with normalization to the reference gene GAPDH. All reactions were performed in triplicate, and at least three independent biological replicates were analyzed.

### 2.9 Cytokine Analysis (ELISA)

The levels of the pro‐inflammatory cytokines interleukin‐6 (IL‐6) and tumor necrosis factor‐alpha (TNF‐α) were quantified in culture supernatants using uncoated human ELISA kits (Invitrogen, Thermo Fisher Scientific). The assay was performed according to the manufacturer's instructions. Briefly, high‐binding 96‐well plates were coated overnight at 4°C with capture antibodies specific for IL‐6 and TNF‐α, diluted in coating buffer. After blocking nonspecific binding sites, samples and standards were added and incubated for 2 h at room temperature. Detection was carried out using biotinylated secondary antibodies followed by streptavidin–HRP and TMB substrate. The reaction was stopped with 1 M sulfuric acid, and absorbance was measured at 450 nm using a Multiskan FC microplate reader (Thermo Fisher Scientific). Cytokine concentrations were determined by interpolation from standard curves generated using known concentrations of recombinant human IL‐6 and TNF‐α and expressed as pg/mL.

### 2.10 Apoptosis Detection (TUNEL Assay) and Caspase 3

Apoptotic cell death was assessed by detecting DNA fragmentation using the In Situ Cell Death Detection Kit (Roche), following the manufacturer's protocol. After treatments, SH‐SY5Y cells were fixed in 4% paraformaldehyde for 20 min at room temperature, then permeabilized with 0.1% Triton X‐100 in 0.1% sodium citrate for 5 min on ice. Cells were incubated with the TUNEL reaction mixture for 1 h at 37°C in a humidified chamber protected from light. After incubation, nuclei were counterstained with DAPI (1 μg/mL) to allow total nuclear visualization. Fluorescent images were acquired using an EVOS M5000 Imaging System (Thermo Fisher Scientific) with appropriate filters. For each condition, at least five random fields per well were imaged at 20× magnification. Quantification of apoptosis was performed by manually counting the number of TUNEL‐positive nuclei and expressing them as a percentage of total DAPI‐stained nuclei using ImageJ software (NIH). The apoptotic index (%) was calculated and plotted as a bar graph using GraphPad Prism 9. Data represent the mean ± SEM from at least three independent experiments performed in triplicate. Caspase‐3 activity, a well‐characterized pro‐apoptotic signal, was assessed using a colorimetric assay kit (CASP‐3‐C, Sigma‐Aldrich) following the manufacturer's instructions. Briefly, the release of p‐nitroaniline (pNA) was measured spectrophotometrically at 405 nm. Results are expressed as fold change relative to control.

### 2.11 Statistical Analysis

All experiments were performed in at least three independent biological replicates, each conducted in technical triplicate. Data are presented as mean ± standard error of the mean (SEM). Statistical analyses were performed using GraphPad Prism version 10 (GraphPad Software, San Diego, CA, USA). One‐way analysis of variance (ANOVA) or Two‐way ANOVA was applied to compare multiple groups, followed by Bonferroni's post hoc test for multiple comparisons. Differences were considered statistically significant at *p* < 0.05.

## 3 Results

### 3.1 FB1 Reduces Cell Viability in a Dose‐Dependent Manner in SH‐SY5Y Cells

To evaluate the cytotoxic effects of FB1 on SH‐SY5Y cells, both undifferentiated and RA‐differentiated cells were exposed to increasing concentrations of FB1 (1, 10, 50, and 100 μM) for 24 h, and cell viability was assessed by MTT assay. As shown in Figure 1A, FB1 induced a mild and non‐linear reduction in cell viability, with no significant effects observed up to 50 μM in either cell type. In undifferentiated cells, a significant decrease in viability was observed at 100 μM, while differentiated cells exhibited a more pronounced sensitivity to FB1. In fact, a highly significant reduction in viability was detected at the same concentration, suggesting that differentiated SH‐SY5Y cells are more vulnerable to FB1‐induced neurotoxicity (Figure 1A). These results are consistent with previous studies showing limited cytotoxicity below 100 μM in neuronal cells [6, 22, 23, 24]. Based on this, 50 μM FB1 was selected for subsequent experiments aimed at investigating early cellular stress responses, including oxidative imbalance, mitochondrial dysfunction, and pro‐inflammatory cytokine release. This concentration was chosen because it induced measurable cellular alterations without triggering overt cytotoxicity, allowing the characterization of mechanistic pathways activated during the sublethal phase of FB1‐induced stress [20]. Furthermore, the use of a subtoxic dose enabled us to evaluate the efficacy of potential neuroprotective agents, such as bromelain, in mitigating early stress responses—a more clinically relevant scenario where functional impairment precedes irreversible cell damage. To rule out any intrinsic cytotoxic or stimulatory effect of the bromelain, SH‐SY5Y cells were treated for 24 h with bromelain alone at 10, 50, and 100 μg/mL. None of the tested concentrations altered basal cell viability in either undifferentiated or RA‐differentiated cells (Figure 1B). These data, consistent with previous reports, confirm that the bromelain had no toxicity or to unspecific metabolic stimulation.

**FIGURE 1:**
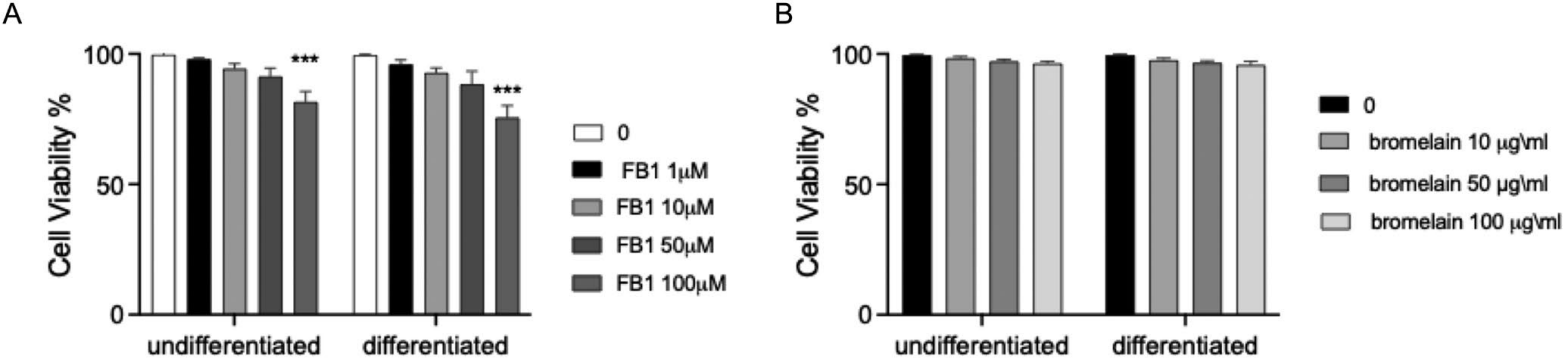
FB1 reduces cell viability in a dose‐dependent manner in SH‐SY5Y cells. (A) Undifferentiated and RA‐differentiated SH‐SY5Y cells were treated with increasing concentrations of FB1 (1, 10, 50, and 100 μM) for 24 h. Cell viability was assessed by MTT assay and is expressed as a percentage relative to untreated control cells (0 μM). Data are presented as mean ± SEM of at least three independent experiments performed in triplicate. (B) Cells were treated for 24 h with bromelain (10, 50 or 100 μg/mL) in the absence of FB1. No reduction in viability was detected. Data represent mean ± SEM; ***p < 0.001; Statistical analysis: One‐way ANOVA + Bonferroni post hoc; *p* = 0.0017 (FB1 100 μM vs. CTR, undifferentiated); *p* = 0.0001 (FB1 100 μM vs. CTR, differentiated).

### 3.2 Bromelain Attenuates FB1‐Induced ROS Production in SH‐SY5Y Cells

To investigate whether the cytoprotective effect of bromelain against FB1 involves modulation of oxidative stress, intracellular ROS levels were measured in SH‐SY5Y cells using the carboxy‐H₂DCFDA probe. As shown in Figure 2, FB1 treatment (50 μM for 24 h) caused a strong increase in ROS generation in both undifferentiated and differentiated cells, with the latter showing a more pronounced response. These findings are consistent with the increased vulnerability of neuron‐like cells to oxidative insults. Fluorescence microscopy (Figure 2A) and flow cytometry analysis (Figure 2B) clearly showed the shift in ROS levels induced by FB1, visible as a stronger green fluorescence signal and a rightward shift in fluorescence intensity, respectively. Quantitative analysis (Figure 2C) confirmed that FB1 significantly elevated ROS production compared to untreated controls, particularly in differentiated cells. Notably, bromelain co‐treatment attenuated the oxidative stress induced by FB1 in a dose‐dependent manner. While 10 μg/mL bromelain moderately reduced ROS levels in differentiated cells, 50 μg/mL bromelain significantly decreased ROS in both cell types, restoring values close to control levels. Together, these data indicate that bromelain effectively mitigates FB1‐induced oxidative stress, supporting its potential role as a protective antioxidant agent in neuronal‐like cells, as summarize in Table S1.

**FIGURE 2:**
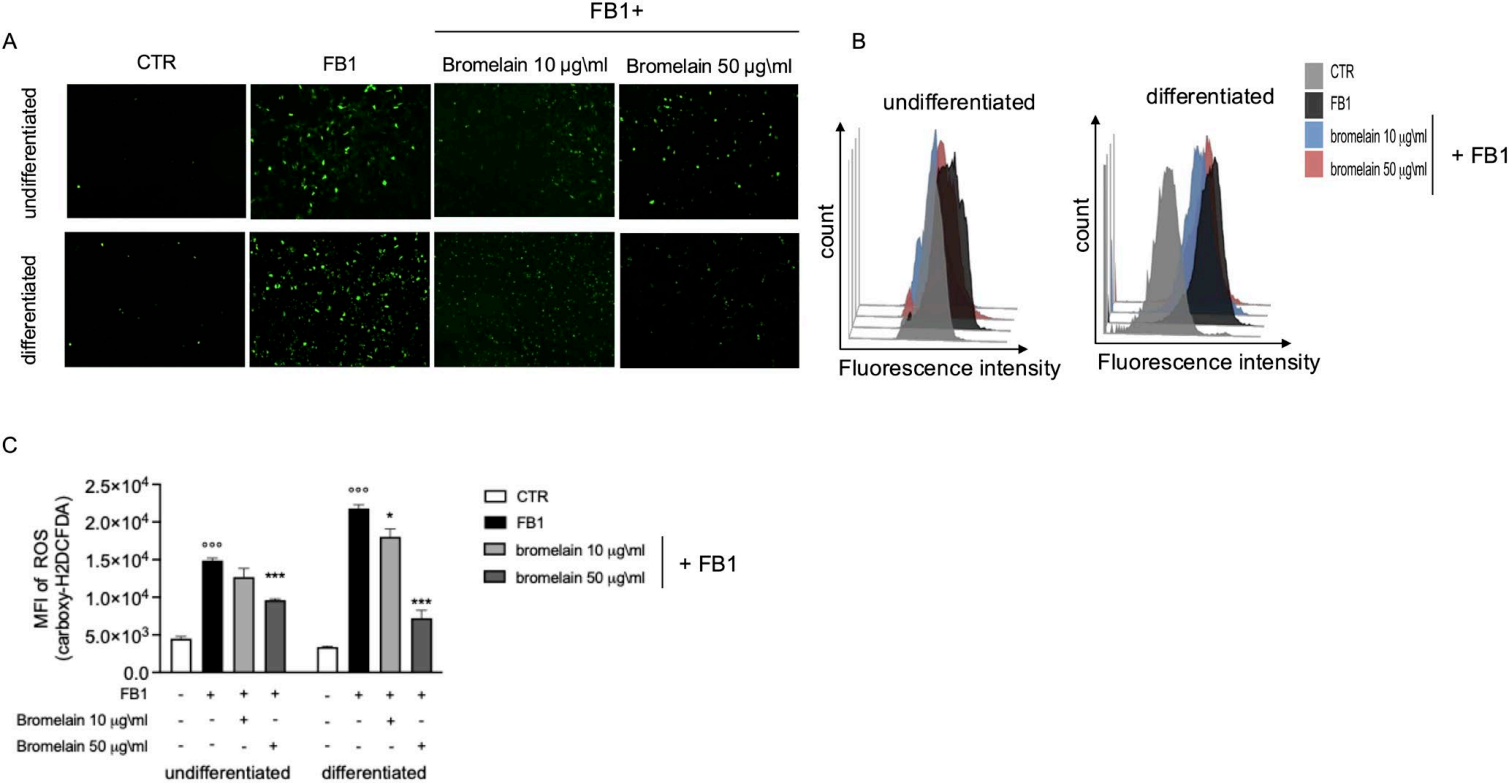
Bromelain attenuates FB1‐induced ROS production in SH‐SY5Y cells. (A) Representative fluorescence microscopy images of intracellular ROS (green signal) detected by carboxy‐H₂DCFDA staining in undifferentiated and RA‐differentiated SH‐SY5Y cells. Cells were treated with FB1 (50 μM) with or without bromelain (10 or 50 μg/mL) for 24 h. (B) Flow cytometry histograms showing fluorescence intensity profiles of carboxy‐H₂DCFDA in each condition. (C) Quantification of mean fluorescence intensity (MFI) of ROS. Data represent mean ± SEM of three independent experiments. °°°*p* < 0.001 vs. CTR; *p < 0.05 vs. FB1; ***p < 0.001 vs. FB1; Statistical analysis: *p* = < 0.0001 (FB1 vs. CTR, undifferentiated); *p* = 0.0001 (FB1 vs. Bromelain 50 μg\mL, undifferentiated); *p* = < 0.0001 (FB1 vs. CTR, differentiated); *p* = 0.0221 (FB1 vs. Bromelain 10 μg\mL, undifferentiated); *p* = < 0.0001 (FB1 vs. Bromelain 50 μg\mL, undifferentiated).

### 3.3 Bromelain Prevents FB1‐Induced Mitochondrial Depolarization in SH‐SY5Y Cells

To determine whether FB1‐induced oxidative stress was associated with mitochondrial dysfunction, we assessed mitochondrial membrane potential (Δψm) using the Rhodamine 123 fluorescent probe. Loss of Δψm—indicated by reduced Rhodamine 123 retention—is a hallmark of early mitochondrial depolarization and cellular stress. As shown in Figure 3, FB1 exposure (50 μM, 24 h) led to a marked increase in mitochondrial depolarization in both undifferentiated and differentiated SH‐SY5Y cells, as evidenced by a leftward shift in fluorescence intensity and a significant increase in the percentage of depolarized cells. Once again, differentiated cells displayed higher sensitivity to FB1, showing greater mitochondrial impairment compared to their undifferentiated counterparts. Importantly, bromelain co‐treatment attenuated FB1‐induced mitochondrial depolarization in a concentration‐dependent manner. Both 10 and 50 μg/mL bromelain significantly reduced the percentage of depolarized cells in FB1‐treated samples, restoring mitochondrial membrane potential closer to control levels. as summarize in Table S1, these data indicate that bromelain exerts a protective effect on mitochondrial function in the context of FB1‐induced toxicity, further supporting its role in mitigating early intracellular stress pathways.

**FIGURE 3:**
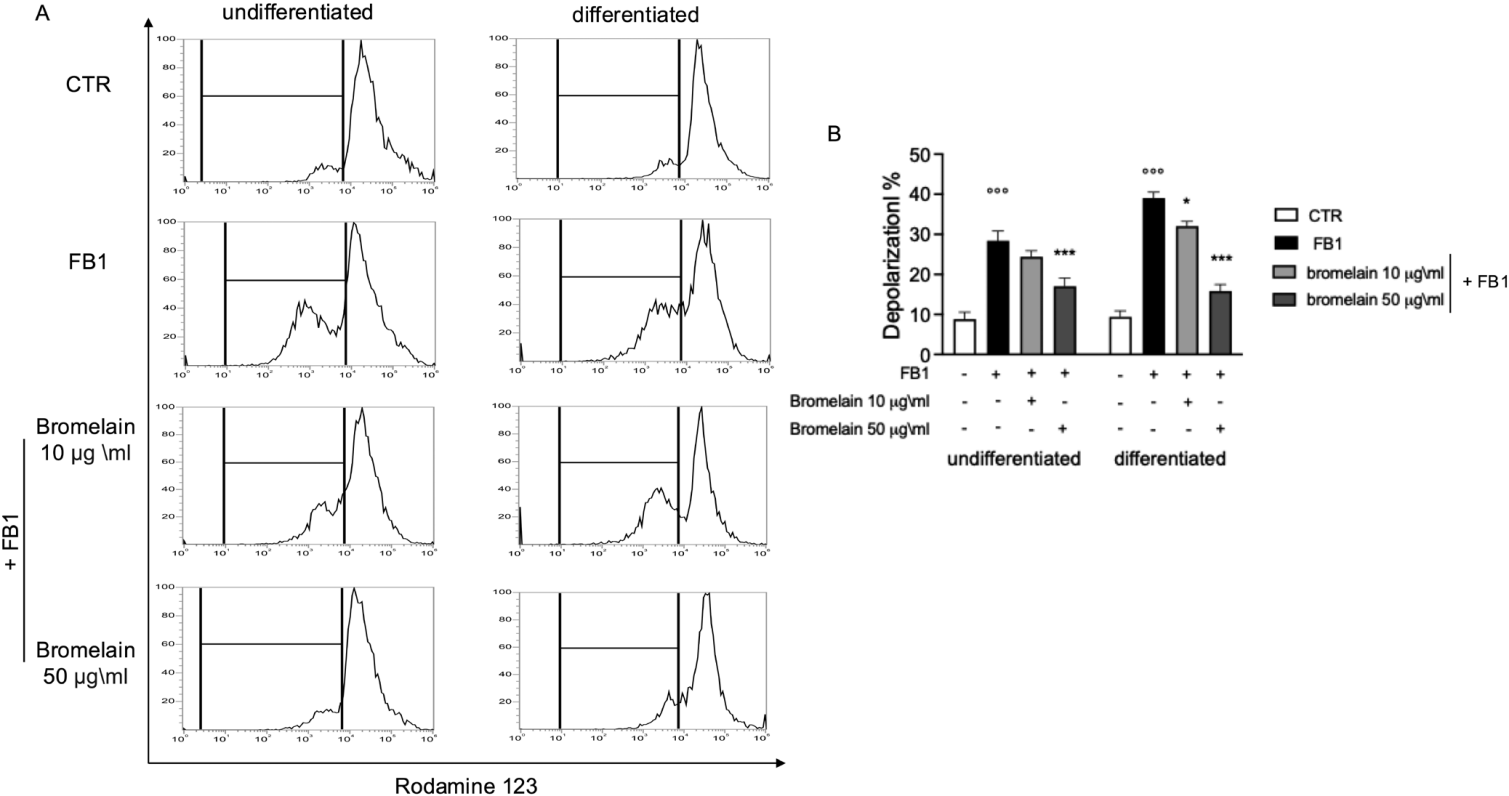
Bromelain prevents FB1‐induced mitochondrial depolarization in SH‐SY5Y cells. Mitochondrial membrane potential (Δψm) was assessed using Rhodamine 123 staining in undifferentiated and RA‐differentiated SH‐SY5Y cells after 24 h treatment with FB1 (50 μM), alone or in combination with bromelain (10 or 50 μg/mL). Left: Representative flow cytometry histograms showing fluorescence intensity shifts in Rhodamine 123 signal. Right: Quantification of mitochondrial depolarization expressed as percentage of cells with reduced Rhodamine 123 fluorescence. Data are reported as mean ± SEM of three independent experiments. °°°*p* < 0.001 vs. CTR, *p < 0.05 vs. FB1, ***p < 0.001 vs. FB1; Statistical analysis: *p* = < 0.0001 (FB1 vs. CTR, undifferentiated); *p* = 0.0002 (FB1 vs. Bromelain 50 μg\mL, undifferentiated); *p* = < 0.0001 (FB1 vs. CTR, differentiated); *p* = 0.0232 (FB1 vs. Bromelain 10 μg\mL, undifferentiated); *p* = < 0.0001 (FB1 vs. Bromelain 50 μg\mL, undifferentiated).

### 3.4 Bromelain Reduces FB1‐Induced Oxidative Stress and Inflammation in SH‐SY5Y Cells

To further explore the impact of FB1 and the protective role of bromelain, we investigated the Nrf2/HO‐1 pathway together with markers of oxidative damage. As shown in Figure 4, exposure to FB1 markedly disrupted the antioxidant response. In differentiated SH‐SY5Y cells, FB1 reduced Nrf2 nuclear translocation, reflected by a decreased nuclear/cytoplasmic (N/C) ratio, and suppressed HO‐1 mRNA expression. These effects were particularly pronounced in differentiated cells, which are metabolically more vulnerable. Remarkably, bromelain co‐treatment restored Nrf2 activation in a dose‐dependent manner, increasing the N/C ratio and significantly upregulating HO‐1 expression, with the 50 μg/mL concentration producing the strongest effect. Consistent with the impairment of antioxidant defenses, FB1 triggered pronounced oxidative stress, as indicated by a significant accumulation of malondialdehyde (MDA), a marker of lipid peroxidation. FB1 treatment significantly increased MDA levels in both undifferentiated and differentiated cells, with differentiated cells again being more sensitive. Co‐treatment with bromelain markedly reduced MDA accumulation in a dose‐dependent manner: the 50 μg/mL dose significantly decreased MDA levels in both cell types, while 10 μg/mL produced a partial protective effect.

**FIGURE 4:**
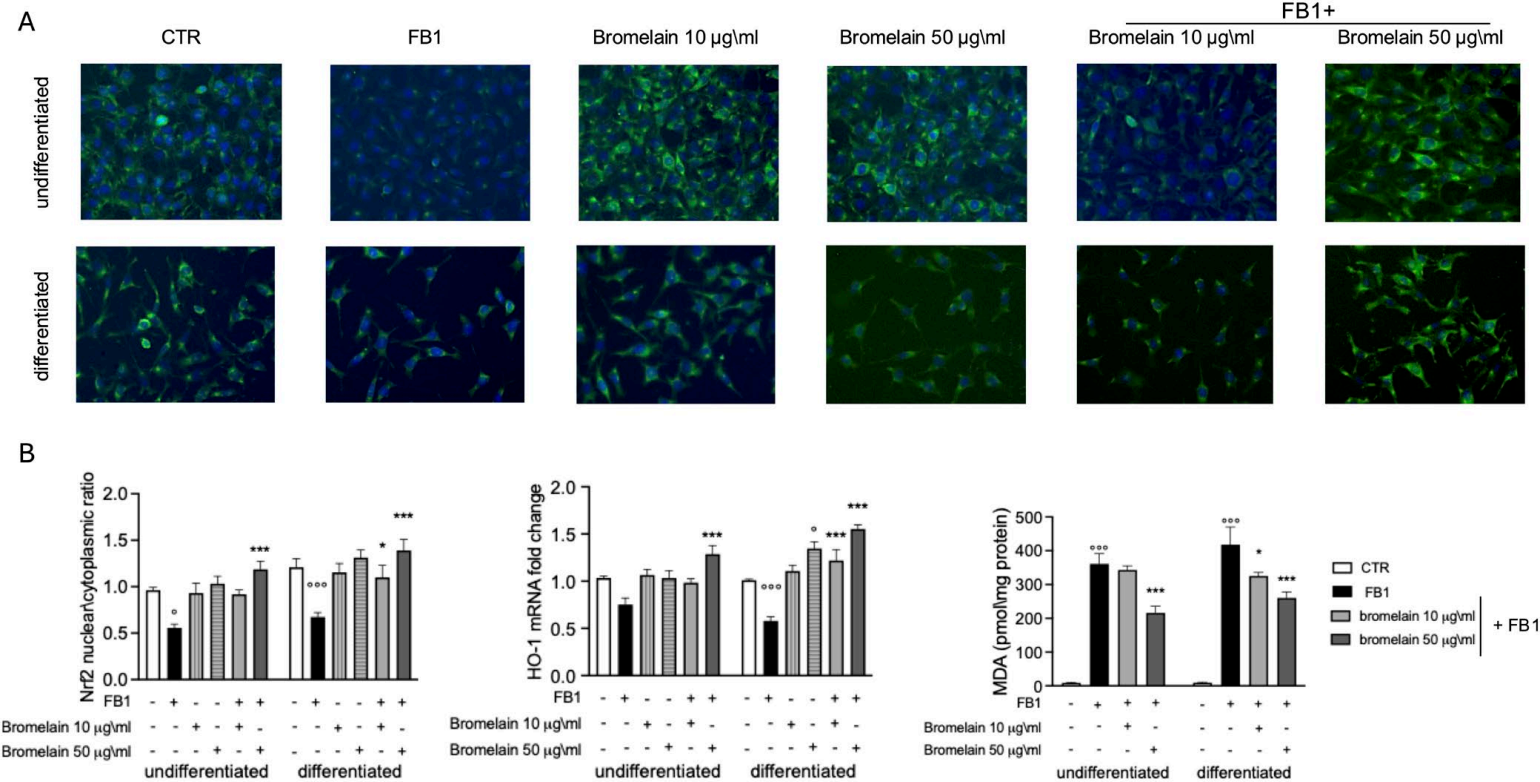
Bromelain reduces FB1‐induced oxidative stress. (A) Representative immunofluorescence images of Nrf2 (green) and nuclei (DAPI, blue) in undifferentiated and RA‐differentiated SH‐SY5Y cells treated with FB1 in the absence or presence of bromelain (10 or 50 μg/mL). (B) Quantification of Nrf2 nuclear/cytoplasmic ratio (left), HO‐1 mRNA expression (middle, normalized to GAPDH, ΔΔ*Ct* method, fold versus CTR), and malondialdehyde (MDA) levels (right, nmol/mg protein). Data are mean ± SEM of three independent experiments. °*p* < 0.05, °°*p* < 0.01, °°°*p* < 0.001 versus CTR; *p < 0.05 versus FB1, **p < 0.01 versus FB1, ***p < 0.001 versus FB1. Statistical analysis: (NRF2) *p* = 0.0244 (FB1 vs. CTR, undifferentiated); *p* = < 0.0001 (FB1 vs. Bromelain 50 μg\mL, undifferentiated); *p* = 0.0008 (FB1 vs. CTR, differentiated); *p* = 0.0142 (FB1 vs. Bromelain 10 μg\mL, differentiated); *p* = < 0.0001 (FB1 vs. Bromelain 50 μg\mL, differentiated); (HO‐1) *p* = < 0.0001 (FB1 vs. Bromelain 50 μg\mL, undifferentiated); *p* = 0.0004 (FB1 vs. CTR, differentiated); *p* = 0.0102 (CTR vs. Bromelain 50 μg\mL, differentiated); *p* = < 0.0001 (FB1 vs. Bromelain 10 μg\mL, differentiated); *p* = < 0.0001 (FB1 vs. Bromelain 50 μg\mL, differentiated); (MDA) *p* = < 0.0001 (FB1 vs. CTR, undifferentiated); *p* = < 0.0005 (FB1 vs. Bromelain 50 μg\mL, undifferentiated; *p* = < 0.0001) (FB1 vs. CTR, differentiated); *p* = 0.0302 (FB1 vs. Bromelain 10 μg\mL, differentiated); *p* = < 0.0002 (FB1 vs. Bromelain 50 μg\mL, differentiated).

Since NF‐κB is a central mediator of pro‐inflammatory signaling, we next examined its activation status in SH‐SY5Y cells. As shown in Figure 5A,B, FB1 robustly increased NF‐κB p65 nuclear translocation in both undifferentiated and RA‐differentiated SH‐SY5Y cells, as indicated by a higher nuclear/cytoplasmic (N/C) ratio. Co‐treatment with bromelain attenuated this effect, but the magnitude depended on the cell phenotype. In undifferentiated cells, 10 μg/mL produced the stronger reduction of the N/C ratio compared with 50 μg/mL, whereas in differentiated cells the 50 μg/mL dose yielded the largest decrease, bringing N/C values close to control. We assayed TNF‐α and IL‐6 secretion, two pro‐inflammatory cytokines under NF‐κB transcriptional control. As shown in Figure 5C, FB1 markedly elevated both TNF‐α and IL‐6 levels in undifferentiated and RA‐differentiated SH‐SY5Y cells. Bromelain significantly blunted this inflammatory response, with a clear dose–response for both cytokines, as summarized in Table S1.

**FIGURE 5:**
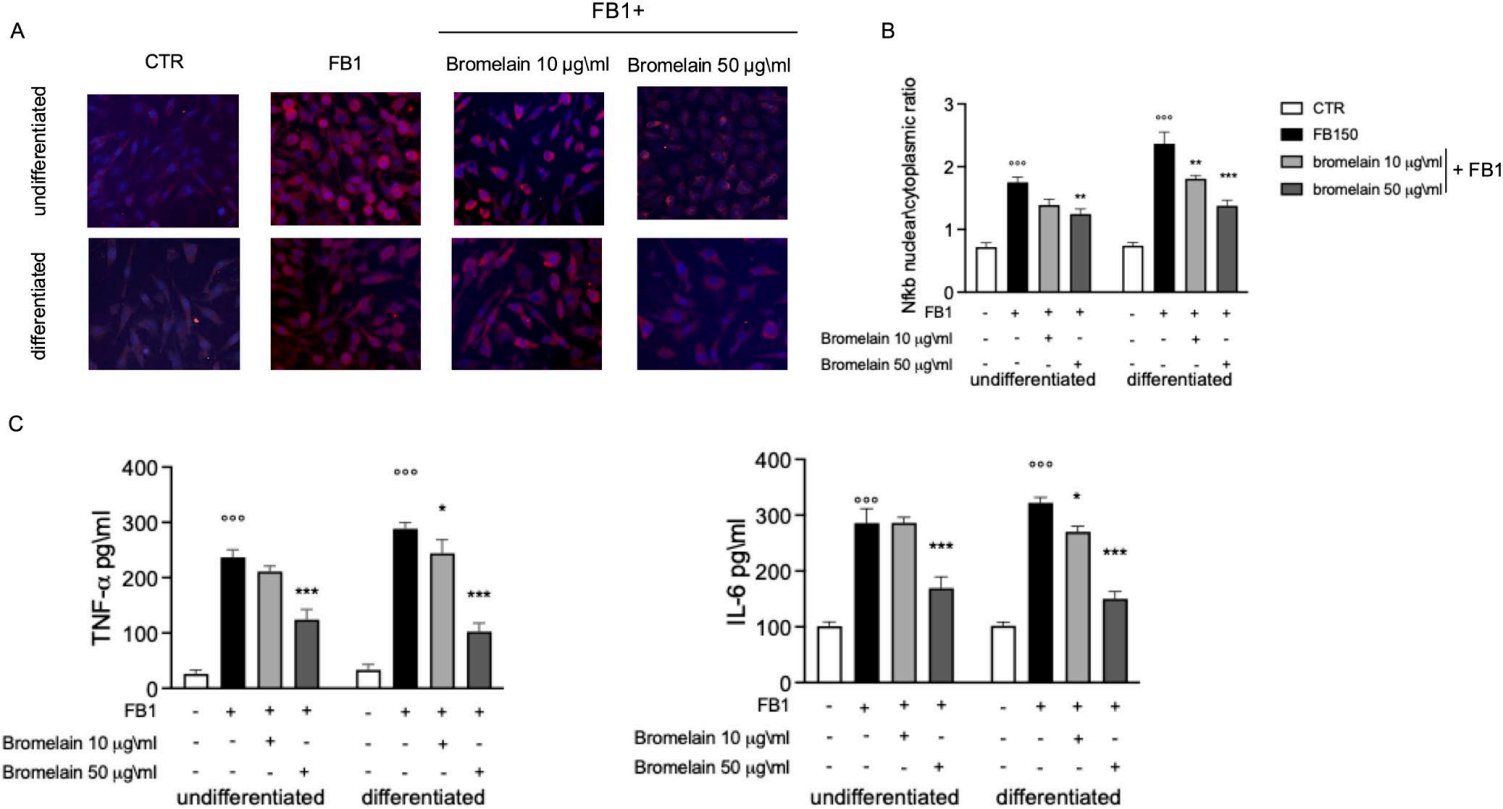
Bromelain attenuates FB1‐induced NF‐κB activation and pro‐inflammatory cytokine release in SH‐SY5Y cells. (A) Representative IF images of NF‐κB p65 (red) and nuclei (DAPI, blue) in undifferentiated and RA‐differentiated cells after with or without bromelain (10 or 50 μg/mL). (B) Quantification of NF‐κB nuclear/cytoplasmic (N/C) ratio. (C) TNF‐α and IL‐6 levels in supernatants (ELISA). Data are mean ± SEM from three independent experiments.°°°*p* < 0.001 vs. CTR; *p < 0.05 vs. FB1, **p < 0.01 vs. FB1, ***p < 0.001 vs. FB1. Statistical analysis: (NF‐κB) *p* = < 0.0001 (FB1 vs. CTR, undifferentiated); *p* = 0.0047 (FB1 vs. Bromelain 50 μg\mL, undifferentiated); *p* = < 0.0001 (FB1 vs. CTR, differentiated); *p* = 0.0016 (FB1 vs. Bromelain 10 μg\mL, differentiated); *p* = < 0.0001 (FB1 vs. Bromelain 50 μg\mL, differentiated); (TNF‐α) *p* = < 0.0001 (FB1 vs. CTR, undifferentiated); *p* = < 0.0001 (FB1 vs. Bromelain 50 μg\mL, undifferentiated); *p* = < 0.0001 (FB1 vs. CTR, differentiated); *p* = 0.0425 (FB1 vs. Bromelain 10 μg\mL, differentiated); *p* = < 0.0001 (FB1 vs. Bromelain 50 μg\mL, differentiated); (IL‐6) *p* = < 0.0001 (FB1 vs. CTR, undifferentiated); *p* = < 0.0001 (FB1 vs. Bromelain 50 μg\mL, undifferentiated); *p* = < 0.0001 (FB1 vs. CTR, differentiated); *p* = 0.0484 (FB1 vs. Bromelain 10 μg\mL, differentiated); *p* = < 0.0001 (FB1 vs. Bromelain 50 μg\mL, differentiated).

### 3.5 Bromelain Protects SH‐SY5Y Cells From FB1‐Induced Apoptosis

To confirm whether the protective effects of bromelain extend to the prevention of FB1‐induced apoptosis, we performed TUNEL staining and caspase‐3 activity assays. As shown in Figure 6A, FB1 exposure markedly increased the number of TUNEL‐positive nuclei, visible as intense magenta fluorescence, particularly in differentiated cells. Quantification (Figure 6B, left) confirmed a significant rise in apoptotic index in both cell types, with differentiated cells showing a stronger apoptotic response. Bromelain co‐treatment significantly reduced DNA fragmentation in a concentration‐dependent manner, with 50 μg/mL providing the most robust protection, and 10 μg/mL affording partial but significant rescue. In line with these findings, FB1 also enhanced caspase‐3 activity, a key effector of apoptosis (Figure 6B, right). Caspase‐3 activity was significantly elevated in both undifferentiated and differentiated cells after FB1 challenge (Figure 6C). Bromelain markedly reduced caspase‐3 activation, with the 50 μg/mL dose lowering activity close to control group, the 10 μg/mL dose exerting partial but significant inhibition. as summarize in Table S1, together, these results demonstrate that bromelain protects SH‐SY5Y cells from FB1‐induced apoptosis by limiting both DNA fragmentation and caspase‐3 activation.

**FIGURE 6:**
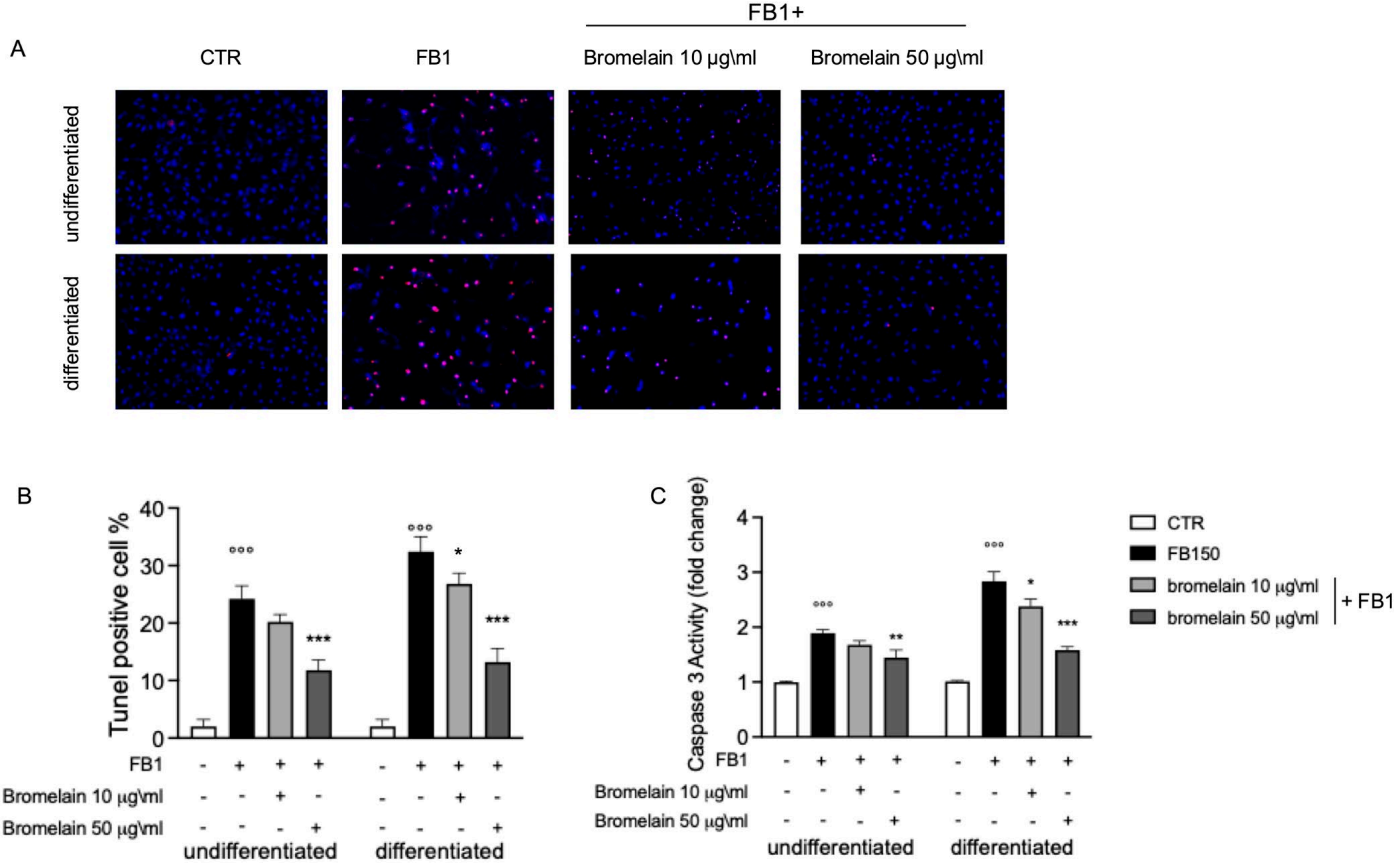
Bromelain protects SH‐SY5Y cells from FB1‐induced apoptosis. (A) Representative fluorescence microscopy images of TUNEL staining in undifferentiated and RA‐differentiated SH‐SY5Y cells. Nuclei are stained with DAPI (blue), and apoptotic nuclei appear TUNEL‐positive (pink). (B) Quantification of TUNEL‐positive cells, expressed as percentage of total nuclei. (C) and caspase‐3 activity (fold change vs. CTR). Data are presented as mean ± SEM. °°°*p* < 0.001 vs. CTR; *p < 0.05, **p < 0.01 vs. FB1, ***p < 0.001 vs. FB1. Statistical analysis: (Tunel) *p* = < 0.0001 (FB1 vs. CTR, undifferentiated); *p* = 0.0004 (FB1 vs. Bromelain 50 μg\mL, undifferentiated); *p* = < 0.0001 (FB1 vs. CTR, differentiated); *p* = 0.0469 (FB1 vs. Bromelain 10 μg\mL, differentiated); *p* = < 0.0001 (FB1 vs. Bromelain 50 μg\mL, differentiated); (Caspase‐3) *p* = < 0.0001 (FB1 vs. CTR, undifferentiated); *p* = 0.001 (FB1 vs. Bromelain 50 μg\mL, undifferentiated); *p* = < 0.0001 (FB1 vs. CTR, differentiated); *p* = 0.0101 (FB1 vs. Bromelain 10 μg\mL, differentiated); *p* = < 0.0001 (FB1 vs. Bromelain 50 μg\mL, differentiated).

## 4 Discussion

### 4.1 FB1 Neurotoxicity: Oxidative and Inflammation

Mycotoxins such as fumonisin B1 (FB1), ochratoxin A (OTA), and zearalenone (ZEA) are increasingly recognized not only for their systemic toxicity but also for their potential effects on the nervous system [25]. In neuronal models, however, the neurotoxic impact of FB1 remains controversial. Several studies have reported limited or no direct effects of FB1 on SH‐SY5Y cells under certain conditions, suggesting that the response may depend on differentiation status, exposure time, or the endpoints measured [26]. Nevertheless, other reports highlight the ability of mycotoxins, including FB1, to disrupt redox homeostasis, lipid peroxidation, and inflammatory signaling, thereby contributing to neuronal dysfunction [27]. In this context, our study extends previous observations by showing that FB1 induces oxidative stress, NF‐κB activation, and apoptotic signaling more strongly in RA‐differentiated SH‐SY5Y cells, a phenotype known to be metabolically more vulnerable. Importantly, we demonstrate that co‐treatment with bromelain counteracts these detrimental effects, in line with its reported antioxidant and anti‐inflammatory activities in neuronal and non‐neuronal models. In line with previous reports, our findings support the notion that the neurotoxic potential of FB1 is not necessarily mediated by a direct cytotoxic effect on neurons, but rather by its ability to trigger oxidative stress and inflammatory signaling cascades [1, 20, 28, 29]. This helps reconcile apparently divergent results in the literature, where some studies reported little or no neuronal loss upon FB1 exposure, while others highlighted alterations in redox balance, lipid peroxidation, or cytokine release [28]. Within this framework, our model based on RA‐differentiated SH‐SY5Y cells allowed us to capture sublethal but functionally relevant responses, including impaired Nrf2/HO‐1 signaling, NF‐κB activation, and apoptosis. These findings suggest that FB1 neurotoxicity may manifest primarily as a dysregulation of redox and inflammatory homeostasis, rather than as overt neuronal cell death, thereby providing a mechanistic basis for bromelain's protective effects. Oxidative stress has also been shown to modulate the inflammatory response, establishing a close and reciprocal relationship between redox imbalance and neuroinflammation. Although these two processes originate from distinct pathological pathways, they are often interconnected and mutually reinforcing, especially in the context of neurodegeneration. Neuropathological research has identified a complex interplay of contributing factors—including environmental exposures, genetic predispositions, physiological alterations, and immune dysregulation—that converge to disrupt neuronal integrity. Among the key mechanisms implicated are oxidative stress, neuroinflammation, and programmed cell death, which together form a central axis in the pathophysiology of neurodegeneration. A deeper understanding of these interconnected pathways is crucial for identifying novel therapeutic targets and improving disease management strategies [30]. Our study contributes extending current knowledge by showing that FB1 triggers oxidative, inflammatory, and apoptotic responses in RA‐differentiated SH‐SY5Y cells, and that bromelain can mitigate these effects, reinforcing the urgency of further investigating its neurological impact within a One Health framework.

### 4.2 Differentiation‐Dependent Effects in SH‐SY5Y Cells

In this context, the use of the SH‐SY5Y neuroblastoma cell line, in both its undifferentiated and retinoic acid (RA)‐differentiated states, provides a valuable in vitro model to assess FB1‐induced neurotoxicity different stages of neuronal development [31]. Undifferentiated SH‐SY5Y cells retain proliferative capacity and resemble immature neuroblasts, whereas RA‐differentiated cells exhibit neuronal‐like morphology and express markers of mature neurons [32]. Our results clearly show that differentiated SH‐SY5Y cells are more susceptible to FB1‐induced damage, highlighting the relevance of using differentiated models to better approximate the response of mature neurons to environmental insults. These findings reinforce growing concerns regarding the broader toxicological profile of FB1, traditionally associated with hepatic and renal toxicity, and highlight its emerging role as a potential environmental neurotoxin. Although bromelain has not been shown to modulate neuronal differentiation markers, future work could assess whether its antioxidant profile indirectly promotes neurogenesis.

### 4.3 Role of Bromelain Against FB1 Toxicity

The rationale for selecting bromelain in this study stems from its well‐documented antioxidant, anti‐inflammatory, and anti‐apoptotic properties, which are increasingly being investigated in both neuronal and non‐neuronal models [33]. In this context, the present work was designed as a proof‐of‐concept investigation, aimed at exploring mechanistic interactions between FB1 toxicity and bromelain's protective signaling in a controlled neuronal model. Bromelain is a proteolytic enzyme complex derived from *Ananas comosus* that has been shown to modulate redox signaling by enhancing endogenous antioxidant defenses and reducing ROS accumulation [34, 35]. In addition, it suppresses NF‐κB–driven inflammatory pathways and cytokine release, thereby attenuating neuroinflammatory cascades [35]. Preclinical studies further indicate that bromelain can exert neuroprotective effects in models of oxidative or excitotoxic injury [36], including SH‐SY5Y cells exposed to 6‐OHDA and rodent models of neurodegeneration [16]. A further caveat that merits discussion concerns the translational relevance of our in vitro bromelain doses. Although we used 10–50 μg/mL in cell culture, the extent to which such concentrations might be achieved in vivo (especially in the brain) is uncertain. Some pharmacokinetic and absorption studies indicate that bromelain can be absorbed in the intestine in its active form (approximately 40% absorption reported) and circulate bound to plasma antiproteases [37]. Oral administration studies in animals and humans have documented a half‐life in plasma on the order of 6–9 h and peak levels ~1 h post‐dose [38]. Nevertheless, the ability of bromelain to cross the blood–brain barrier and reach effective concentrations in neuronal tissue remains incompletely characterized [36]. We thus acknowledge this as a limitation and propose that future in vivo studies should carefully assess bromelain pharmacokinetics, BBB permeability, and dose–response correlation in CNS tissues. In our study, FB1 exposure led to a significant increase in intracellular ROS levels, particularly in RA‐differentiated SH‐SY5Y cells, suggesting greater vulnerability of neuron‐ like cells to oxidative injury.

### 4.4 FB1 Pathways

Bromelain treatment effectively reduced ROS production in a dose‐dependent manner, confirming its antioxidant potential. Because mitochondria are a major source of ROS generated during oxidative phosphorylation, mitochondrial dysfunction is tightly linked to oxidative stress in neurodegenerative contexts [36]. Under physiological conditions, mitochondria are equipped with enzymatic and non‐enzymatic antioxidant systems that maintain redox homeostasis. To better understand the redox imbalance induced by FB1, we therefore investigated mitochondrial integrity. FB1 exposure resulted in a marked loss of mitochondrial membrane potential (Δψm), as assessed by Rhodamine 123 staining, indicating early mitochondrial stress and identifying mitochondria as primary targets of FB1‐mediated toxicity [39]. Δψm depolarization was selected as a sensitive and validated early marker of mitochondrial impairment, which precedes overt bioenergetic failure. Although ATP production or oxygen consumption rate (OCR) measurements would provide complementary information, Δψm loss represents an established proxy for early mitochondrial dysfunction. A central finding of this study concerns the modulation of the Nrf2 pathway. FB1 markedly reduced Nrf2 nuclear translocation, particularly in RA‐differentiated SH‐SY5Y cells, coinciding with increased ROS generation and lipid peroxidation (MDA). These results suggest that FB1 impairs endogenous antioxidant defenses while promoting oxidative damage. In contrast, bromelain restored Nrf2 nuclear accumulation and upregulated HO‐1 expression, concomitantly reducing ROS and MDA levels. This supports the notion that bromelain‐mediated neuroprotection is closely linked to the preservation of Nrf2‐driven antioxidant responses [39], positioning Nrf2 as a key regulator of the balance between oxidative injury and cellular protection during FB1 exposure. In parallel, FB1 robustly activated the NF‐κB pathway, as evidenced by increased nuclear translocation of the p65 subunit and elevated secretion of the pro‐inflammatory cytokines TNF‐α and IL‐6. These effects were more pronounced in RA‐differentiated SH‐SY5Y cells, consistent with their higher susceptibility to toxic and inflammatory stimuli. Bromelain significantly attenuated both NF‐κB activation and cytokine release, with the higher concentration exerting the strongest inhibitory effect. This dual modulation of oxidative and inflammatory pathways suggests that bromelain can disrupt the feed‐forward loop linking oxidative stress to downstream inflammatory signaling. Such oxidative–inflammatory crosstalk is a key driver of neurodegenerative progression, in part through activation of resident immune cells within the central nervous system, particularly microglia [40]. Sustained neuroinflammation is known to amplify pro‐apoptotic signaling cascades mediated by inflammatory cytokines and redox imbalance [41]. In line with this framework, FB1 exposure induced significant apoptotic responses, as demonstrated by increased DNA fragmentation (TUNEL assay) and caspase‐3 activation. Importantly, bromelain co‐treatment markedly reduced both apoptotic indices, confirming its ability to suppress apoptosis in addition to its antioxidant and anti‐inflammatory actions. Collectively, these findings indicate that bromelain preserves redox balance, limits inflammatory signaling, and prevents apoptosis in both undifferentiated and RA‐differentiated SH‐SY5Y cells, supporting its potential as a broadly applicable neuroprotective agent against FB1‐induced neuronal stress.

### 4.5 Experimental Limitations and Future Research Perspectives

Taken together, our findings position bromelain as a promising candidate for neuroprotection against FB1 toxicity. The present study was intentionally designed to capture early mitochondrial stress responses, using mitochondrial membrane potential (Δψm) as a sensitive and well‐established indicator that precedes overt bioenergetic failure. While functional bioenergetic readouts such as ATP production and oxygen consumption rate (OCR) would provide complementary information, Δψm assessment is particularly suited to detect early mitochondrial perturbations induced by toxic insults. Future studies integrating Δψm with direct bioenergetic measurements will allow a more comprehensive characterization of FB1‐induced mitochondrial dysfunction. Although upstream signaling pathways such as MAPKs and PI3K/Akt are known modulators of Nrf2 and NF‐κB activity, the present study focused on downstream functional outcomes associated with FB1‐induced neurotoxicity, including oxidative stress, mitochondrial dysfunction, inflammation, and apoptosis. Future mechanistic studies will be required to delineate the contribution of these upstream kinase pathways to bromelain‐mediated neuroprotection. Given that MAPKs (ERK, JNK, p38) and PI3K/AKT are recognized modulators of both Nrf2 and NF‐κB activity, future studies should clarify whether bromelain exerts its protective effects through the regulation of these upstream kinases. By disrupting the oxidative stress–inflammation–apoptosis axis, bromelain helps maintain redox balance, preserve mitochondrial integrity, and enhance cellular resilience to environmental stressors. Its natural origin, low toxicity, and current nutraceutical use further strengthen its translational potential. Although bromelain stability and intracellular availability were not directly assessed in the present study, its biological activity under the experimental conditions is supported by the consistent, dose‐dependent modulation of multiple independent cellular endpoints. Future studies integrating pharmacokinetic and cellular uptake analyses will be important to further define bromelain stability, bioavailability, and subcellular distribution in neuronal systems. While bromelain's ability to cross the blood–brain barrier remains debated, reports indicate limited penetration of proteolytic fragments into CNS tissue following systemic administration [34]. This limitation should be acknowledged, and in vivo pharmacokinetic and biodistribution analyses are warranted to confirm translational relevance Considering the widespread occurrence of fumonisins in the food chain and their emerging neurological implications, natural compounds such as bromelain may represent a practical and accessible strategy to mitigate mycotoxin‐related neurodegenerative risk, meriting further evaluation in preclinical and in vivo models. Given bromelain's pleiotropic antioxidant capacity, its efficacy against other neurotoxic mycotoxins such as ochratoxin A or zearalenone deserves future investigation.

## 5 Conclusions

In summary, our results demonstrate that fumonisin B1 (FB1) exerts measurable adverse effects in SH‐SY5Y cells primarily through oxidative stress, lipid peroxidation, mitochondrial depolarization, inflammatory cytokine release, and activation of apoptotic pathways. Importantly, apoptosis was confirmed by both DNA fragmentation (TUNEL assay) and caspase‐3 activity, supporting the role of FB1 in promoting neuronal injury. Differentiated SH‐SY5Y cells, which more closely mimic mature neurons, were particularly susceptible, highlighting that FB1 neurotoxicity may become more evident in metabolically active neuronal phenotypes. These observations are consistent with previous studies reporting that, while FB1 does not always induce overt neuronal loss, it can compromise redox balance and inflammatory homeostasis, thereby contributing indirectly to neurotoxicity. Within this framework, our study identifies bromelain as a promising protective agent. Bromelain mitigated FB1‐induced oxidative stress, restored Nrf2/HO‐1 signaling, reduced NF‐κB activation and cytokine release, and ultimately prevented apoptosis. Its capacity to act on multiple stress pathways simultaneously, combined with its natural origin, low toxicity, and established nutraceutical use, supports its translational potential. Although our work was conducted in vitro, and further validation in in vivo models is required to establish pharmacokinetics, blood–brain barrier penetration, and dose relevance, these findings suggest that bromelain could represent a practical strategy to counteract the neurological risks posed by dietary fumonisin exposure.

## Author Contributions

Conceptualization: E.G. Methodology, and Formal Analysis: D.D.P, F.I., and G.A.F. Investigation: N.T. Resources: S.C. Data curation: R.F. Writing – original draft preparation: F.I. and G.A.F. All authors have read and agreed to the published version of the manuscript.

## Funding

The authors have nothing to report.

## Ethics Statement

The authors have nothing to report.

## Conflicts of Interest

The authors declare no conflicts of interest.

## Supporting information


**Data S1:** Supporting Information Tables.

## Data Availability

The data that support the findings of this study are available from the corresponding author upon reasonable request.
