# Supplementary material for: Environmental Mycotoxins and Brain Health: Protective Role of Bromelain Against Fumonisin B1 in SH‐SY5Y Cells
Source: Environ Toxicol. 2026 Feb 24;41(11):1188–200. doi: 10.1002/tox.70059 (PMC13656879; doi:10.1002/tox.70059)
Supplement: Supplementary file 1 [file TOX-41-1188-s001.docx]

**Table 1.** ***Bromelain attenuates FB1-induced ROS production in SH-SY5Y cells*. Fold change vs control**

|  | CTR | FB1 | FB1+ Bromelain 10μg\ml | FB1+ Bromelain 50μg\ml |
| --- | --- | --- | --- | --- |
| undifferentiated | 1 | 3, | 3,3 | 3,3 |
| differentiated | 1 | 4,8 | 4,8 | 4,8 |

**Table 2** **Fold change vs control. Bromelain prevents FB1-induced mitochondrial depolarization in SH-SY5Y cells.**

|  | CTR | FB1 | FB1+ Bromelain 10μg\ml | FB1+ Bromelain 50μg\ml |
| --- | --- | --- | --- | --- |
| undifferentiated | 1 | 3,2 | 2,7 | 1,9 |
| differentiated | 1 | 4,1 | 3,5 | 1,6 |

**Table 3** **Fold change vs control. Bromelain reduces FB1-induced oxidative stress**

(MDA)

|  | CTR | FB1 | FB1+ Bromelain 10μg\ml | FB1+ Bromelain 50μg\ml |
| --- | --- | --- | --- | --- |
| undifferentiated | 1 | 41 | 36,9 | 24,5 |
| differentiated | 1 | 47,5 | 39,9 | 29,5 |

**Table 4** **Fold change vs control. Bromelain attenuates FB1-induced NF-κB activation and pro-inflammatory cytokine release in SH-SY5Y cells.**

TNF-α

|  | CTR | FB1 | FB1+ Bromelain 10μg\ml | FB1+ Bromelain 50μg\ml |
| --- | --- | --- | --- | --- |
| undifferentiated | 1 | 9,1 | 8,1 | 4,7 |
| differentiated | 1 | 8,7 | 7,3 | 3,1 |

IL-6

|  | CTR | FB1 | FB1+ Bromelain 10μg\ml | FB1+ Bromelain 50μg\ml |
| --- | --- | --- | --- | --- |
| undifferentiated | 1 | 10,9 | 11 | 6,5 |
| differentiated | 1 | 9,7 | 8,1 | 4,5 |

**Table 5** **Fold change vs control. Bromelain protects SH-SY5Y cells from FB1-induced apoptosis.**

tunel

|  | CTR | FB1 | FB1+ Bromelain 10μg\ml | FB1+ Bromelain 50μg\ml |
| --- | --- | --- | --- | --- |
| undifferentiated | 1 | 12,1 | 10,1 | 5,9 |
| differentiated | 1 | 16,2 | 13,4 | 6,6 |

caspase-3 activity

|  | CTR | FB1 | FB1+ Bromelain 10μg\ml | FB1+ Bromelain 50μg\ml |
| --- | --- | --- | --- | --- |
| undifferentiated | 1 | 1,8 | 1,6 | 1,4 |
| differentiated | 1 | 2,8 | 2,3 | 1,5 |
